# Age- and Sex-Specific Social Contact Patterns and Incidence of *Mycobacterium tuberculosis* Infection

**DOI:** 10.1093/aje/kwv160

**Published:** 2015-12-08

**Authors:** Peter J. Dodd, Clare Looker, Ian D. Plumb, Virginia Bond, Ab Schaap, Kwame Shanaube, Monde Muyoyeta, Emilia Vynnycky, Peter Godfrey-Faussett, Elizabeth L. Corbett, Nulda Beyers, Helen Ayles, Richard G. White

**Keywords:** disease burden, infection incidence, social contact pattern, tuberculosis

## Abstract

We aimed to model the incidence of infection with *Mycobacterium tuberculosis* among adults using data on infection incidence in children, disease prevalence in adults, and social contact patterns. We conducted a cross-sectional face-to-face survey of adults in 2011, enumerating “close” (shared conversation) and “casual” (shared indoor space) social contacts in 16 Zambian communities and 8 South African communities. We modeled the incidence of *M. tuberculosis* infection in all age groups using these contact patterns, as well as the observed incidence of *M. tuberculosis* infection in children and the prevalence of tuberculosis disease in adults. A total of 3,528 adults participated in the study. The reported rates of close and casual contact were 4.9 per adult per day (95% confidence interval: 4.6, 5.2) and 10.4 per adult per day (95% confidence interval: 9.3, 11.6), respectively. Rates of close contact were higher for adults in larger households and rural areas. There was preferential mixing of close contacts within age groups and within sexes. The estimated incidence of *M. tuberculosis* infection in adults was 1.5–6 times higher (2.5%–10% per year) than that in children. More than 50% of infections in men, women, and children were estimated to be due to contact with adult men. We conclude that estimates of infection incidence based on surveys in children might underestimate incidence in adults. Most infections may be due to contact with adult men. Treatment and control of tuberculosis in men is critical to protecting men, women, and children from tuberculosis.

Tuberculosis remains a major global public health problem. Approximately 1 in 3 people globally might be infected with *Mycobacterium tuberculosis* and at risk of progressing to tuberculosis disease (1). In 2014, there were approximately 9.6 million new tuberculosis disease cases and 1.5 million deaths (2).

Despite the huge global burden of tuberculosis disease, there are important gaps in the understanding of patterns of *M. tuberculosis* transmission. The primary mechanism of *M. tuberculosis* infection is inhaling droplets containing bacilli aerosolized by an infectious tuberculosis case (1), but how *M. tuberculosis* infection incidence varies by age, particularly among adults, and the characteristics of the source cases are poorly known.

The incidence of *M. tuberculosis* infection by age can be estimated using direct methods that rely on the follow-up of initially uninfected individuals over time or indirect methods that typically rely on statistical analysis of data on the prevalence of *M. tuberculosis* infection (3). Direct estimates from Canada, South India, the United States, and Malawi suggest that the incidence rate of *M. tuberculosis* infection is higher in adults (4–8), but this has been attributed in part to the instability of the tuberculin skin test (TST) (4). Historic decisions on the upper age limits in TST prevalence surveys have meant that estimates using indirect methods in persons older than 20 years of age are rare (9, 10). The limited data that do exist from TST prevalence surveys suggest that infection incidence increased with increasing age into adulthood in Uganda and Saskatchewan but decreased in South Africa (11–13). As such, data on the incidence of *M. tuberculosis* infection among adults are limited and equivocal.

There is also substantial uncertainty about where *M. tuberculosis* transmission takes place. Sustained household exposure has traditionally been the assumed route of transmission. More recently, molecular and other data have suggested a substantial role for transmission outside the household (14–19) and led to investigations in indoor settings in the community, where people congregate (20, 21). Mathematical models of the transmission of acute respiratory infections are sensitive to assumptions about contact patterns between different age groups (22–24). This has led to diary and interview-based attempts to empirically measure and analyze social mixing patterns in Europe (25–32), Asia (33), and more recently, South Africa (34).

Understanding infection incidence by age and the characteristics of source cases is critical to informing control programs. In the absence of reliable estimates of *M. tuberculosis* infection incidence among adults, we performed a survey of social contact patterns and modeled the age- and sex-specific *M. tuberculosis* infection incidence and the sexes of source cases. We combined social contact data, data on the incidence *M. tuberculosis* infection among schoolchildren, and data on the prevalence of adult tuberculosis from 24 communities in Zambia and South Africa (35–37).

## METHODS

### Ethics statement

Ethics approval was obtained from University of Stellenbosch (N04/10/173) Health Research Ethics Committee, the University of Zambia Biomedical Research Ethics Committee (007-10-04), and the London School of Hygiene and Tropical Medicine Ethics Committee (A211 3008).

### Social contact survey

Adults (≥18 years of age) enrolled in the Zambia-South Africa TB and AIDS Reduction (ZAMSTAR) Study (35) final tuberculosis prevalence survey that was carried out in 2010 in 16 communities in Zambia and 8 communities in the Western Cape, South Africa, were randomly selected for face-to-face interviews. Interviews took place during daylight hours in February and March 2011 in Zambia and in May and July 2011 in South Africa. Four ZAMSTAR standard enumeration areas (SEAs) in each community were randomly selected proportional to size, and within each SEA, 10 individuals were randomly selected from 4 age and sex strata: men 18–29 years of age, men ≥30 years of age, women 18–29 years of age, and women ≥30 years of age (160 per community). Individuals were not eligible if they had not spent the previous night in the SEA. If an individual was ineligible, did not consent, or was not found after 2 visits, another individual was randomly selected from the same stratum in that SEA.

Interviews were carried out in participants' homes using a standardized questionnaire. The questionnaire was piloted in Zambia in early 2011 based on insights from participatory research carried out in both countries in 2005 (21). Interviewees were asked about their age, sex, number of cohabitees (hereafter referred to as household size), and their recent contact history. Interviewees were asked to report contacts that occurred in the 24 hours preceding the midnight before the interview. Two types of contact were measured: “close” and “casual” contacts. A close contact was defined as contact with someone with whom the interviewee had a face-to-face conversation that was longer than a greeting and within an arm's reach. Information was gathered on the age and sex of each person contacted, the place and duration of the contact (see below), and the frequency of contact with this person. Casual contacts were defined as contacts with people who were inside buildings other than the interviewee's home that the interviewee had visited. Interviewees were asked to report close contacts with individuals of all ages, as well as the number of casual contacts with individuals 5–12 years of age and 13 years of age or older, over the previous 24 hours. The study questionnaire is included in Web Appendix 1 (available at http://aje.oxfordjournals.org/).

### Location and duration of contacts

The durations and locations of close and casual contacts were recorded. Casual contact locations were chosen following the social science surveys in both countries in 2005–2006 and in Zambia in 2011 (21, 38) (see Web Appendix 2 for detail of locations).

### Data analysis

Data were double-entered into an SQL server database and analyzed using Microsoft Excel 2011 (39), Stata (40), and R (41). The contact rate for a given type of contact was defined as the mean number of individuals contacted by each adult per day. Unadjusted and adjusted mean contact rates were calculated to identify differences in contact rates between communities as a whole, accounting for the sample design. Adjusted mean contact rates were calculated by weighting data on individuals for community population size and the age and sex proportions in the SEA (see Web Appendix 2 for weighting methodology). The number of casual contacts was based on the category midpoint (excepting the “>20” category, for which 21 contacts was assumed) and modeled with a zero-inflated negative binomial distribution (42). Contact rates were examined by interviewee age category, sex, household size, day of the week, urban or rural community, and setting. Analyses were repeated for close and casual contacts and contacts with children (≤12 years of age).

The duration of casual contact for each interviewee was defined as the sum of the reported time spent in each location multiplied by the number of casual contacts reported there. Duration category midpoints were used.

### Estimated incidence of *M. tuberculosis* infection

We estimated the age- and sex-specific incidence rate of *M. tuberculosis* infection (λ*_i_*), where the index *i* = 1, …, 5 represents female participants 0–4, 5–12, 13–25, 26–45, and ≥46 years of age, respectively, and *i* = 6, …, 10 represents male participants of the corresponding ages. These age groups were chosen to approximately match the ages for which we have data on the incidence of *M. tuberculosis* infection in children (≈5–12 years of age) and the groups we interviewed in our contact study. We use index α to denote the groups interviewed in our contact study, where α = 1,2,3 for women 18–25, 26–45, and ≥46 years old, respectively, and α = 4,5,6 for men 18–25, 26–45, and ≥ 46 years old, respectively. We let β_α*i*_ be the rate at which individuals in a group α come into effective contact with individuals in group *i* and *p*_α_ be the per capita prevalence of culture-positive individuals in group α. Assuming mass-action mixing within groups, we arrive at equation 1 below. In equation 2, we further assume that the effective contact rate is proportional (with coefficient β_0_) to the corresponding close contact rates measured in our survey (*c*). In equation 3, we further assume that the total numbers of contacts per unit time between 2 groups (*i* and α) are symmetrical, so that *N*_α_*c*_α*i*_ = *N_i_c_i_*_α_, where *N_i_* and *N*_α_ are the number of individuals in groups *i* and α, respectively. For example, the total number of contacts per unit time reported by men 18–25 years of age with women 26–45 years of age is equal to the total number of contacts per unit time reported by women 26–45 years of age with men 18–25 years of age. The *c*_α*i*_ were estimated directly from our contact data.

(1) $$\lambda_{i}=\overset{6}{\underset{\alpha =1}{\sum }}\beta_{i\alpha }\;p_{\alpha }\;i=1,\;\ldots ,10$$

(2) $$\;\lambda_{i}=\overset{6}{\underset{\alpha =1}{\sum }}\beta_{0}c_{i\alpha }\;p_{\alpha }$$

(3) $$\begin{array}{l} =\overset{6}{\underset{\alpha =1}{\sum }}\beta_{0}\left(\frac{N_{\alpha }c_{\alpha i}}{N_{i}}\right)\;p_{\alpha } \end{array}$$

In these communities, the prevalence of culture-positive tuberculosis disease in adults (>18 years of age) was measured in the 2011 ZAMSTAR final prevalence survey (35) (giving *p*_α_), and the annual risk of *M. tuberculosis* infection in children 5–12 years old was estimated from a TST survey in 2005 to be 4.2% per year in Western Cape and 1.2% per year in Zambia (37). We used this incidence rate of *M. tuberculosis* infection that was empirically measured in children (37) to set the constant of proportionality (β_0_) in the model and thus to predict infection rates in older age groups. The relative infectiousness of smear-positive versus smear-negative culture tuberculosis disease cases was ignored because a similar proportion of culture positive cases were also smear-negative by country, and this overall proportion would be absorbed into β_0_ on scaling to the incidence of *M. tuberculosis* infection in children. We used estimates of the annual risk of *M. tuberculosis* infection based on the mixture method (37), which might be more robust to differences in the prevalence of nontuberculous mycobacteria between countries than are estimates based on other methods. Confidence intervals were computed from 10^4^ bootstraps assuming the numbers of contacts were Poisson-distributed and the demographic proportions were Dirichlet-distributed. The demographic populations in equation 3 were from the household enumeration part of our study and are given in the Web Table 1. Sensitivity of results to mixing is investigated in Web Appendix 3.

## RESULTS

### Participants

Of the 5,875 eligible individuals sought, 14% could not be located on 2 attempts, 23% had moved, 1% had died, 2% refused to participate, and 60% consented; therefore, 3,528 interviewees participated in the survey (Table 1). This represented 3% (3,528 of 123,790) of adults enumerated in the ZAMSTAR final prevalence survey. A total of 1,831 (52%) were female and 2,256 (64%) were in Zambia; 1,176 (33%), 1,489 (42%), and 863 (25%) were 18–25 years old, 26–45 years old, and ≥46 years old, respectively. Interviewees in Zambia reported larger households than did those in South Africa (mean number of people per household = 4.6, 95% confidence interval (CI): 4.5, 4.7, and 3.6, 95% CI: 3.5, 3.7, respectively), and fewer South African households than Zambian households included a 5–12-year-old child (40% versus 60%; *P* < 0.001).

**Table 1.** Characteristics of Social Contact Survey Interviewees in 16 Communities in Zambia and 8 Communities in Western Cape, South Africa, 2011

Interviewee Characteristic	Zambia (*n* = 2,256)		Western Cape, South Africa (*n* = 1,272)		Total (*n* = 3,528)	
	No.	%	No.	%	No.	%
Age of interviewee, years						
18–25	779	34.5	397	31.2	1,176	33.3
26–45	911	40.4	578	45.4	1,489	42.2
≥46	566	25.1	297	23.4	863	24.5
Sex of interviewee						
Female	1,193	52.9	638	50.2	1,831	51.9
Male	1,063	47.1	633	49.8	1,696	48.1
Missing			1	0.1	1	0.0
Years lived in household						
<6 months	73	3.2	12	0.9	85	2.4
6–11 months	109	4.8	8	0.6	117	3.3
1–4 years	613	27.2	225	17.7	838	23.8
5–9 years	395	17.5	303	23.8	698	19.8
≥10 years	1,022	45.3	709	55.7	1,731	49.1
Missing	44	2.0	15	1.2	59	1.7
Years lived in community						
<6 months	14	0.6	3	0.2	17	0.5
6–11 months	59	2.6	5	0.4	64	1.8
1–4 years	419	18.6	159	12.5	578	16.4
5–9 years	366	16.2	273	21.5	639	18.1
≥10 years	1,343	59.5	819	64.4	2,162	61.3
Missing	55	2.4	13	1.0	68	1.9
No. of people in household^a,b^	4.6	4.5, 4.7	3.6	3.5, 3.7	4.3	4.2, 4.4
No. of children in household						
0	860	38.1	763	60.0	1,623	46.0
1	545	24.2	355	27.9	900	25.5
2	423	18.8	119	9.4	542	15.4
3	222	9.8	27	2.1	249	7.1
4	70	3.1	3	0.2	73	2.1
≥5	39	1.7	5	0.4	44	1.3
Missing	97	4.3	0	0.0	97	2.8
No. of children 5–12 years old in household^a,c^	1.2	1.1, 1.2	0.6	0.5, 0.6	1.0	0.9, 1.0
No. of hours spent inside the previous day (including sleeping)^a,d^	14.0	13.8, 14.3	17.8	17.5, 18.1	15.4	15.2, 15.6

Abbreviation: CI, confidence interval.

^a^ Values are expressed as means and 95% confidence intervals.

^b^ Data were missing for 134 interviewees.

^c^ Data were missing for 97 interviewees.

^d^ Data were missing for 255 interviewees.

### Close contacts

The 3,528 interviewees reported 17,451 close contacts with persons of all other ages in the preceding 24 hours (Table 2). Fifteen percent (2,695 contacts) were with 0–12-year-old children. The adjusted rate of close contact was 4.9 (95% CI: 4.6, 5.2) contacts per adult per day; the rate for close contact with 0–12-year-old children was 0.8 (95% CI: 0.7, 0.9).

**Table 2.** Close Contact Rate by Interviewee Characteristic in 16 Communities in Zambia and 8 Communities in Western Cape, South Africa, 2011

Interviewee Characteristic	Contacts With Persons of All Ages						Contacts With Children 0–12 Years Old Only					
	No. of Contacts	No. of Interviewees	Unadjusted Contact Rate per Adult per Day^a^		Adjusted Contact Rate per Adult per Day^a^		No of Contacts	No. of Interviewees^b^	Unadjusted Contact Rate per Adult per Day^c^		Adjusted Contact Rate per Adult per Day^c^	
			Mean	95% CI	Mean	95% CI			Mean	95% CI	Mean	95% CI
All interviewees	17,451	3,528	4.9	4.8, 5.0	4.9	4.6, 5.2	2,695	3,426	0.8	0.7, 0.8	0.8	0.7, 0.9
Age of interviewee, years												
18–25	6,014	1,176	5.1	4.9, 5.3	5.0	4.5, 5.4	787	1,146	0.7	0.6, 0.7	0.7	0.6, 0.9
26–45	7,399	1,489	5.0	4.8, 5.1	4.9	4.5, 5.2	1,339	1,447	0.9	0.9, 1.0	0.9	0.8, 1.1
≥46	4,038	863	4.7	4.5, 4.9	4.7	4.4, 5.1	569	833	0.7	0.6, 0.8	0.7	0.6, 0.8
Sex of interviewee												
Female	9,101	1,831	5.0	4.8, 5.1	4.9	4.5, 5.3	1,711	1,767	1.0	0.9, 1.0	0.9	0.8, 1.1
Male	8,346	1,696	4.9	4.8, 5.1	4.8	4.4, 5.2	982	1,658	0.6	0.5, 0.6	0.6	0.5, 0.7
Missing	4	1	4.0	N/A	N/A	N/A	2	1	2.0	N/A	N/A	N/A
Household size												
1	1,043	319	3.3	3.0, 3.5	3.2	2.8, 3.5	40	310	0.1	0.1, 0.2	0.1	0.1, 0.2
2	2,036	526	3.9	3.7, 4.0	3.9	3.6, 4.2	221	516	0.4	0.4, 0.5	0.4	0.3, 0.5
3	2,715	627	4.3	4.1, 4.5	4.3	3.9, 4.6	413	614	0.7	0.6, 0.7	0.7	0.6, 0.8
4	2,984	583	5.1	4.9, 5.3	5.0	4.6, 5.4	454	570	0.8	0.7, 0.9	0.8	0.6, 0.9
5	2,515	447	5.6	5.3, 5.9	5.8	5.3, 6.3	487	433	1.1	1.0, 1.3	1.2	1.0, 1.5
6	1,844	323	5.7	5.4, 6.1	5.8	5.2, 6.3	401	313	1.3	1.1, 1.4	1.3	1.1, 1.5
7	1,363	216	6.3	5.9, 6.8	6.4	5.7, 7.0	233	205	1.1	1.0, 1.3	1.3	0.9, 1.6
8	1,132	171	6.6	6.1, 7.2	6.6	5.8, 7.3	202	168	1.2	1.0, 1.4	1.2	0.9, 1.6
9	1,409	182	7.7	7.0, 8.5	7.8	6.9, 8.6	232	177	1.3	1.0, 1.6	1.4	1.0, 1.8
Missing	410	134	3.1	2.6, 3.5	2.7	2.2, 3.3	12	120	0.1	0.0, 0.2	0.1	0.0, 0.1
Day of the week												
Sunday	3,129	637	4.9	4.7, 5.2	4.8	4.3, 5.3	493	622	0.8	0.7, 0.9	0.8	0.7, 1.0
Monday	3,438	693	5.0	4.7, 5.2	4.7	4.1, 5.2	546	676	0.8	0.7, 0.9	0.8	0.6, 1.0
Tuesday	3,409	684	5.0	4.8, 5.2	5.1	4.7, 5.4	574	657	0.9	0.8, 1.0	0.9	0.8, 1.0
Wednesday	3,317	644	5.2	4.9, 5.4	5.2	4.7, 5.7	488	630	0.8	0.7, 0.9	0.8	0.7, 1.0
Thursday	3,192	665	4.8	4.6, 5.0	4.7	4.1, 5.3	494	652	0.8	0.7, 0.9	0.8	0.6, 1.0
Friday	451	103	4.4	3.8, 5.0	4.9	3.1, 6.7	37	94	0.4	0.2, 0.6	0.5	0.1, 0.9
Saturday	515	102	5.0	4.4, 5.7	5.1	4.0, 6.2	63	95	0.7	0.5, 0.9	0.8	0.2, 1.4
Urban or rural community												
Urban	15,740	3,232	4.9	4.8, 5.0	4.8	4.5, 5.2	2,352	3,132	0.8	0.7, 0.8	0.8	0.7, 0.9
Rural	1,711	296	5.8	5.4, 6.2	5.6	5.3, 6.0	343	294	1.2	1.0, 1.3	1.0	0.7, 1.4
Setting												
Zambia	10,787	2,256	4.8	4.6, 4.9	4.6	4.2, 5.1	1,447	2,159	0.7	0.6, 0.7	0.7	0.5, 0.8
Western Cape, South Africa	6,664	1,272	5.2	5.1, 5.4	5.2	4.8, 5.7	1,248	1,267	1.0	0.9, 1.0	1.0	0.9, 1.2

Abbreviations: CI, confidence interval; N/A, not applicable.

^a^ Excluding 1 interviewee (with 4 close contacts) for whom a sampling weight could not be calculated because information on sex was not available. Therefore, values are based on data from 3,527 interviewees who reported 17,347 close contacts.

^b^ Excluding 102 interviewees for whom information about close contacts with children 0–12 years of age was not available.

^c^ Excluding 1 interviewee (with 2 close contacts with children 0–12 years of age) for whom a sampling weight could not be calculated because information on sex was unavailable.

Adjusted rates of close contact with all ages showed little difference by interviewee age, sex, or day of the week (Table 2). There was strong evidence of higher contact rates in communities with larger households (*P* for trend < 0.001) and in rural communities. Adjusted rates of close contact with children 0–12 years old were higher for participants 26–45 years old, women, residents of larger households, and those in South Africa (Table 2).

### Casual contacts

Data on casual contacts were available for 93% (3,277) of interviewees, who reported 38,128 contacts with persons 5 years of age or older (Table 3). Of these, 34% (12,779) were with children who were 5–12 years of age. The overall adjusted casual contact rate was 10.4 (95% CI: 9.3, 11.6) per adult per day and for contact with children 5–12 years old was 3.5 (95% CI: 3.1, 4.0).

**Table 3.** Casual Contact Rate by Interviewee Characteristics in 16 Communities in Zambia and 8 Communities in Western Cape, South Africa, 2011

Interviewee Characteristic	Contacts With Children ≥5 Years of Age						Contacts With Children 5–12 Years of Age Only					
	No. of Contacts	No. of Interviewees^a^	Unadjusted Contact Rate per Adult per Day^b^		Adjusted Contact Rate per Adult per Day^b^		No. of Contacts	No. of Interviewees^c^	Unadjusted Contact Rate per Adult per Day^d^		Adjusted Contact Rate per Adult per Day^d^	
			Mean	95% CI	Mean	95% CI			Mean	95% CI	Mean	95% CI
All interviewees	38,128	3,277	11.6	11.1, 12.2	10.4	9.3, 11.6	12,779	3,309	3.9	3.6, 4.1	3.5	3.1, 4.0
Age of interviewee, years												
18–25	13,542	1,081	12.5	11.5, 13.6	10.8	9.2, 12.3	4,297	1,095	3.9	3.5, 4.3	3.4	2.8, 4.1
26–45	16,921	1,394	12.1	11.3, 13.0	10.8	9.3, 12.4	5,866	1,404	4.2	3.8, 4.6	3.8	3.2, 4.4
≥46	7,665	802	9.6	8.5, 10.6	8.8	7.1, 10.4	2,616	810	3.2	2.8, 3.7	3.0	2.4, 3.5
Sex of interviewee												
Female	17,573	1,722	10.2	9.4, 11.0	9.5	8.2, 10.7	6,507	1,733	3.8	3.4, 4.1	3.5	3.0, 4.0
Male	20,555	1,554	13.2	12.4, 14.0	12.1	10.3, 13.9	6,272	1,575	4.0	3.7, 4.3	3.6	3.0, 4.1
Missing	0	1	0.0	N/A	N/A	N/A	0	1	0.0	N/A	N/A	N/A
Household size												
1	3,676	305	12.1	10.4, 13.7	11.6	9.5, 13.8	1,112	306	3.6	3.0, 4.3	3.6	2.8, 4.4
2	5,675	490	11.6	10.1, 13.0	10.2	8.1, 12.2	1,878	494	3.8	3.2, 4.4	3.4	2.6, 4.2
3	6,235	574	10.9	9.6, 12.2	8.7	7.3, 10.2	2,138	582	3.7	3.1, 4.2	3.0	2.3, 3.6
4	6,369	550	11.6	10.2, 12.9	11.0	9.2, 12.8	2,203	555	4.0	3.4, 4.5	3.8	3.1, 4.5
5	4,883	410	11.9	10.4, 13.5	11.6	9.4, 13.7	1,600	414	3.9	3.2, 4.5	3.9	3.0, 4.7
6	2,983	309	9.7	8.0, 11.3	9.4	6.6, 12.2	998	311	3.2	2.5, 3.9	3.1	1.9, 4.2
7	2,612	198	13.2	10.5, 15.9	11.4	7.9, 14.9	850	201	4.2	3.2, 5.3	3.7	2.3, 5.1
8	1,809	150	12.1	9.1, 15.0	9.0	6.3, 11.8	675	154	4.4	3.2, 5.6	3.4	2.0, 4.8
9	2,500	164	15.2	12.2, 18.3	13.7	9.7, 17.7	863	164	5.3	4.0, 6.6	4.9	3.4, 6.3
Missing	1,386	127	10.9	8.3, 13.6	9.9	7.3, 12.5	462	128	3.6	2.5, 4.7	2.9	2.0, 3.9
Day of the week												
Sunday	8,419	600	14.0	12.5, 15.5	11.9	9.7, 14.0	3,309	605	5.5	4.8, 6.2	4.7	3.6, 5.7
Monday	6,543	646	10.1	9.0, 11.3	9.4	7.3, 11.4	2,088	655	3.2	2.7, 3.6	2.9	2.2, 3.5
Tuesday	6,621	630	10.5	9.3, 11.7	9.5	7.7, 11.4	2,147	635	3.4	2.9, 3.9	3.2	2.5, 3.8
Wednesday	7,196	590	12.2	10.8, 13.6	10.6	8.8, 12.5	2,273	597	3.8	3.3, 4.3	3.3	2.6, 3.9
Thursday	6,667	619	10.8	9.6, 11.9	10.4	8.7, 12.1	2,112	623	3.4	2.9, 3.9	3.4	2.8, 4.1
Friday	1,260	96	13.1	9.4, 16.8	10.1	7.3, 12.8	387	97	4.0	2.5, 5.5	3.4	2.1, 4.7
Saturday	1,422	96	14.8	11.2, 18.4	13.2	7.3, 19.1	463	97	4.8	3.4, 6.1	4.2	2.0, 6.5
Urban or rural community												
Urban	35,426	3,011	11.8	11.2, 12.4	10.5	9.3, 11.8	11,843	3,040	3.9	3.7, 4.1	3.5	3.1, 4.0
Rural	2,702	266	10.2	8.5, 11.9	8.7	6.5, 10.9	936	269	3.5	2.8, 4.2	3.0	2.1, 3.8
Setting												
Zambia	26,331	2,032	13.0	12.2, 13.7	11.6	10.1, 13.1	9,057	2,057	4.4	4.1, 4.7	4.0	3.4, 4.6
Western Cape, South Africa	11,797	1,245	9.5	8.7, 10.2	8.9	7.2, 10.5	3,722	1,252	3.0	2.7, 3.3	2.9	2.3, 3.4

Abbreviations: CI, confidence interval; N/A, not applicable.

^a^ Excluding 251 interviewees for whom the number of casual contacts was not available.

^b^ Excluding 1 interviewee (with 0 casual contacts) for whom a sampling weight could not be calculated because information on sex not available. Therefore, values are based on 3,276 interviewees who reported 38,128 casual contacts.

^c^ Excluding 219 interviewees for whom number of casual contacts with children 0–12 years of age was not available.

^d^ Excluding 1 interviewee (with 0 casual contacts 0–12 years of age) for whom a sampling weight could not be calculated because information on sex was not available.

Adjusted rates of casual contact with adults and persons of all ages did not show strong evidence of an association with interviewee characteristics (Table 3). The mean community rate of casual contact with children (5–12 years of age) was higher in Zambia than in South Africa (in Zambia, rate = 4.0, 95% CI: 3.4, 4.6; in South Africa, rate = 2.9, 95% CI: 2.3, 3.4) and higher on Sundays than on most other days (Table 3).

### Location and duration of contacts

Most close contacts were reported to have occurred at home (in Zambia, 51%, 95% CI: 47, 55; in South Africa, 73%, 95% CI: 70, 78) (Web Figure 1A). Close contacts were also reported to have occurred in other homes, outside, in work buildings, and in schools (12%, 9%, 5%, and 3%, respectively). Excluding participants' own homes, a higher proportion of close contact time was spent in work buildings than was suggested by the proportion of close contacts in work buildings (25% of contact duration but 15% of contacts) because of longer duration contacts at work (Web Figure 1B).

Of reported casual contacts (Web Figure 1C), 19% occurred in church, 16% in shops, 15% in the interviewee's work building, and 12% in other homes (combined percentages for both settings not shown). Comparing the proportions of casual contacts and casual contact duration by location shows shorter contact episodes in shops, churches, and bars than at work or school (Web Figure 1D).

### Mixing by age and sex

**Figure 1.**
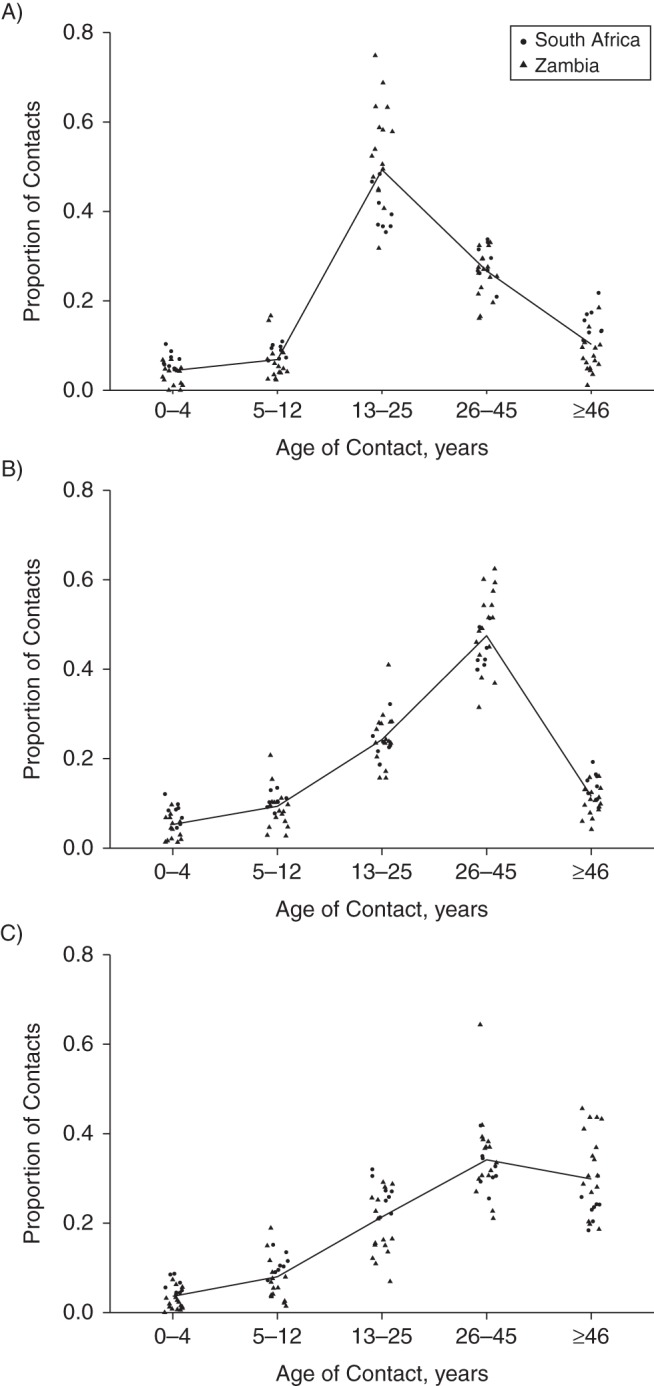
Proportion of close contacts, by contact age and setting, for interviewees aged 18–25 years (A), 26–45 years (B), and ≥46 years (C), Zambia and the Western Cape, South Africa, 2011. Each point is the mean for a community; lines join overall means.

The age distribution of close contacts reported by interviewees indicated strong mixing within age groups (Web Table 2 and Figure 1). There was also strong evidence of within-sex preferential mixing: 63% (5,614 of 8,928) of female interviewees' close contacts and 61% (4,935 of 8,083) of male interviewees' close contacts were reported to be of the same sex as the interviewee (*P* < 0.001). Web Table 2 shows the percentage of close contacts of interviewees who were 18–25, 26–45, and ≥46 years old with male and female contacts by age.

### Estimated incidence of *M. tuberculosis* infection

**Figure 2.**
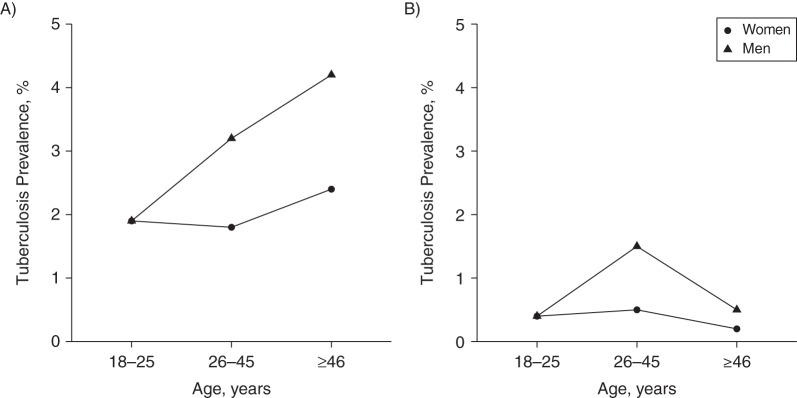
Prevalence of culture positive tuberculosis disease among adults in the Western Cape, South Africa (A) and Zambia (B) from the 2011 Zambia-South Africa TB and AIDS Reduction Study final prevalence survey (35), shown by age, sex and setting. This is used as a model input for estimating *Mycobacterium tuberculosis* infection rates in adults.

The prevalence of culture-positive tuberculosis disease was higher in males than in females in both settings (for females vs. males: in Zambia, 0.4% vs. 0.9%; in South Africa, 2.0% vs 3.0%). The prevalence of culture-positive tuberculosis disease by age, sex, and setting is shown in Figure 2.

**Figure 3.**
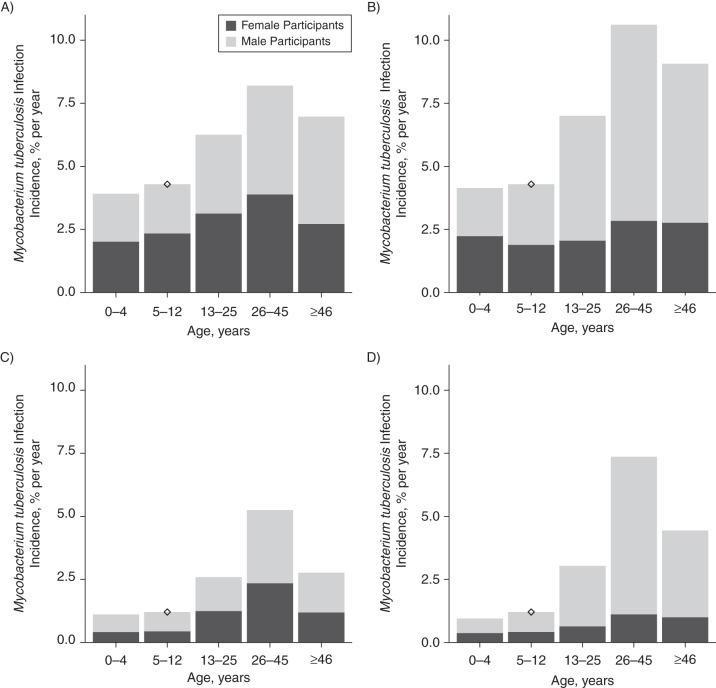
Estimated incidence of *Mycobacterium tuberculosis* infection in women (A and C) and men (B and D) by age and sex of infectious person, Western Cape, South Africa (A and B) and Zambia (C and D). Diamonds show the *M. tuberculosis* infection incidence estimated directly from tuberculin skin test sensitivity data among schoolchildren. Data from Shanaube et al. (37).

The infection incidence was estimated to be 6%–8% and 7%–10% per year for adult (≥13 years of age) females and males, respectively, in South Africa and 2.5%–5% and 3%–7% per year, respectively, in Zambia (Figure 3). The incidence was 1.5–6 times higher than what was empirically measured in children. This ratio increased with the age assortativity in mixing (Web Figure 2). The estimated overall percentage of infections due to contact with adult men was 57.3% (95% CI: 56.3, 58.2) in Western Cape and 65.7% (64.4, 66.8) in Zambia (Web Table 3); it was 50% or higher in all age groups, in both sexes, and in both settings except in 0–12-year-old girls and 0–4-year-old boys in Western Cape.

## DISCUSSION

This work represents the first multicountry quantitative study of social mixing patterns in sub-Saharan Africa, and it was based on a large sample drawn from multiple communities in each country. Our results suggest that rates of close contact with persons of all ages were higher for adults in larger households and in rural areas, whereas overall casual contact rates did not vary by interviewee characteristics. Rates of close contact with children were higher among interviewees who were 26–45 years old, women, adults in larger households, and from South African communities, whereas rates of casual contact with children were higher in interviewees from Zambian communities. There was strong evidence of preferential mixing for close contacts within age groups and within sexes. Our results suggest that the estimated incidence of *M. tuberculosis* infection in adults might be 1.5–6 times higher than what was empirically measured in children because of higher rates of contact between adults and cases with infectious tuberculosis disease, usually other adults. Our observation of within-sex mixing may amplify exposure among men, who have a higher prevalence of the disease. Our results suggest that more than 50% of all infections might be due to contact with adult men.

Our finding that the incidence of *M. tuberculosis* infection might be higher in adults than in children is consistent with previous direct and indirect estimates from Canada, South India, the United States, Malawi, Uganda, and Saskatchewan (4–8, 12, 13) but not a recent indirect estimate from South Africa (11). Our ratio of adult-to-child *M. tuberculosis* infection incidence of 1.5–6 compared with a median ratio of 1.5 (interquartile range, 1.1–7) in those other studies for which comparison was possible. Therefore, it may be that infection rates are often higher in adults than in children, despite concerns about TST instability (4). Our finding that most infections in these communities were due to contact with men was largely because tuberculosis prevalence was higher in male interviewees, and it might be generalizable to other populations because the prevalence tuberculosis disease tends to be higher in men (43). The high proportion of *M. tuberculosis* infection incidence due to contact with men was particularly surprising in young children in Zambia because contact rates with women were higher, which suggests that even in this age group, the higher prevalence in males tended to outweigh the higher contact rates between young children and women. The higher estimated proportion of tuberculosis infections due to men in Zambia is due to the higher relative concentration of tuberculosis disease in men in Zambia.

A potential limitation of the present study was that reported contact rates were lower than rates found in other studies (30, 33, 34). Poor recall and interview fatigue might have led to smaller numbers of contacts being recorded in our study. However, only the relative contact rates, not the absolute contact rates, were used to determine our results regarding *M. tuberculosis* infection incidence, and similar patterns of more intensive mixing within age groups have been observed in other contact studies (30, 34), which supports our conclusions.

Our choice to base the calculation of the incidence of *M. tuberculosis* infection on close contact rates reflects the general uncertainty over what constitutes an effective contact for *M. tuberculosis* transmission and the lower quality of our data on casual contacts. Our analysis suggests that most close contact time was spent within people's homes. Historical studies in developed settings found a higher prevalence of TST positivity among contacts who were members of the case's household (44), and being the spouse of a tuberculosis case was a strong risk factor for *M. tuberculosis* infection in contemporary Malawi (45). However, whether a contact is sufficient for transmission is likely to depend on the duration of contact, volume of contacts, ventilation in the room, and host and pathogen characteristics (1). Further analysis of data on contact patterns combined with measures of tuberculosis disease burden and infection may help identify key locales and activities associated with *M. tuberculosis* transmission and clarify which contacts are more likely to facilitate transmission (46). Our main conclusions depend on mixing within age groups of effective contacts (Web Appendix 3); this is supported by an analysis of DNA fingerprinting data from the Netherlands in which the authors concluded that tuberculosis cases preferentially transmitted infection to people close to their own ages (47).

Another limitation was that data on the prevalence of tuberculosis disease were only available on individuals older than 18 years of age. Some tuberculosis disease cases will have been due to infection from persons younger than 18 years. However, the risk of *M. tuberculosis* infection developing into the infectious forms of tuberculosis disease is much lower in persons younger than 15 years of age than in those who are older (48). Because the proportion of the population 15–17 years of age is very small, transmission from this group is unlikely to affect our main conclusions.

It is possible that individuals with active tuberculosis might have different contact patterns than the rest of the population. We were not able to investigate this directly because sputum was not taken at interview, and the number of interviewees known to have been prevalent cases in the preceding prevalence survey was too small for meaningful comparison. However, the fact the typical duration of active tuberculosis disease exceeds the typical reported duration of symptoms at diagnosis and the substantial fraction of asymptomatic tuberculosis found in prevalence surveys (49) suggest that active tuberculosis may not strongly influence behavior during much of its infectious period. It remains an interesting possibility that individuals with tuberculosis have systematically different contact patterns that put them at greater risk of tuberculosis exposure and infection in the first place. Such heterogeneities might have important implications for transmission, and longitudinal or case-control studies could inform this issue.

It is uncertain how our results may generalize to settings with a different prevalence of human immunodeficiency virus (HIV). Although an HIV-infected individual may be more susceptible to infection/progression, this increased susceptibility applies equally to exposures from different subgroups and should therefore not affect the proportion of infection due to each. However, our results on proportions of infections due to each group may be sensitive to HIV-infected individuals having different contact patterns. In settings with a lower HIV prevalence, one might expect a larger contribution of men to the proportion of *M. tuberculosis* infections because HIV prevalence is typically higher among women than among men in settings with a high HIV prevalence.

We followed Sutherland's definition of the incidence of *M. tuberculosis* infection as the annual rate of infection with tubercle bacilli among individuals who had never previously been infected. (3). However, rates of *M. tuberculosis* infection among never-infected adults might be lower than we estimated. Individuals who remain uninfected at older ages may differ biologically or behaviorally from already-infected individuals; for example, they might benefit from higher innate immunological protection and/or less frequent contact with tuberculosis cases due to preferential mixing by characteristics other than age and sex (e.g., by socioeconomic status). Generalizing our results to other adults, *M. tuberculosis* reinfection rates (infection rates in already-infected adults) may be lower or higher than our estimated infection rates for never-infected adults, for example, because of protection conferred by existing latent infection (which would tend to decrease infection incidence) or more frequent contact with tuberculosis cases (which would tend to increase infection incidence).

Our results suggest that mixing within age groups implies that estimates of *M. tuberculosis* infection incidence based on surveys in children might underestimate infection incidence in adults, and most infections may be due to contact with adult men. For hyperendemic communities in South Africa, which may have an incidence of *M. tuberculosis* infection in children as high as 4% per year (11, 37, 50), our findings imply that *M. tuberculosis* infection incidence rates in never-infected adults may be as high as 10% per year, rates which have rarely been seen outside institutional settings. Care and control of tuberculosis in males is critical to protecting men, women, and children from tuberculosis.

## Supplementary Material

Web Material
