# Supplementary material for: Age- and Sex-Specific Social Contact Patterns and Incidence of Mycobacterium tuberculosis Infection
Source: Am J Epidemiol. 2015 Dec 8;183(2):156–66. doi: 10.1093/aje/kwv160 (PMC4706676; doi:10.1093/aje/kwv160)
Supplement: Web Material [file supp_183_2_156__index.html]

Age- and Sex-Specific Social Contact Patterns and Incidence of Mycobacterium tuberculosis Infection — Web Material 

# Age- and Sex-Specific Social Contact Patterns and Incidence of *Mycobacterium tuberculosis* Infection

## Web Material

Web Material

- Web Material - Pdf file
