# Supplementary material for: Age- and Sex-Specific Social Contact Patterns and Incidence of Mycobacterium tuberculosis Infection
Source: Am J Epidemiol. 2015 Dec 8;183(2):156–66. doi: 10.1093/aje/kwv160 (PMC4706676; doi:10.1093/aje/kwv160)
Supplement: Web Material [file supp_kwv160_kwv160supp.pdf]

GENERAL INFORMATION-- ONLY BEGIN IF CONSENT GIVEN

**Individual barcode**	

00COD	**Interviewer's code**				

		D D	M M	Y Y Y Y	
01DAT	Date of Interview				
02STT	**Start time**				

		F	M
03SEX	**Gender**	0	1

HOUSEHOLD QUESTIONS

04AGE	**How old are you?**					
	**Number** [99 for don't know, 98 for refused]:					
05LOH	**How long have you lived in this household?**					
	**less than 6 months**	**6 months to less than 1 year**	**1 – 4 years**	**5 – 9 years**	**10 or more years**	**Don't know**
	0	1	2	3	4	99
06LOC	**How long have you lived in [*interviewer*:** *say community name***]?**					
	**less than 6 months**	**6 months to less than 1 year**	**1 – 4 years**	**5 – 9 years**	**10 or more years**	**Don't know**
	0	1	2	3	4	99
07HTI	**How many hours did you spend inside your house from midnight yesterday to midnight last night (including sleeping)?**					
	**less than 5 hrs**	**5-8**	**9-13**	**14-19**	**more than 20 hrs**	**Don't know**
	0	1	2	3	4	99

08HRE	**I want to ask about the people who live in your household. By household I mean those you normally eat with, and who slept in your household since [*interviewer: say current time*] yesterday. Please do not include yourself.**

**Household member**	**Age (years)**	**Sex (M/F)**	**Relationship to you**
HRE1			
HRE2			
HRE3			
HRE4			
HRE5			
HRE6			
HRE7			
HRE8			
HRE9			
HRE10			
HRE11			
HRE12			
HRE13			
HRE14			
HRE15			
HRE16			
HRE17			
HRE18			

Key

Code	Relationship
1	Husband/wife
2	Parent
3	Child
4	Grandparent
5	Grandchild
6	Brother/sister
7	Other(related)
8	Other(unrelated)
99	Don't know
98	Refused

09NKI	**[Interviewer:** Write down how many children 5-12 years old live in this household**]**	

10NSR	**How many rooms does your household sleep in?**	
	**Number [99 for don't know, 98 refused]**	

11NWI	**How many windows that open (or that are always open) does your household have?**	
	**Number [99 if don't know, 98 refused]:**	

CHILDREN aged 5-12 years
[SKIP TO ACTIVITIES SECTION,19TVO, IF NO CHILDREN 5-12 YEARS LIVING IN HOUSEHOLD] I would like to ask you about the children living here. By child, I mean someone aged between 5 and 12 years. You said you had [***interviewer****: say number of children aged 5-12 from* NKI] children aged 5-12 years in your household.

12ACS	**How many of these [*interviewer:* *say number*] children aged 5-12 sleep in the same room with an adult?**	
	**Number [99 for don't know, 98 refused]:**	

13NKS	**How many of the children aged 5-12 years old regularly sleep in another household?**	
	**Number [99 for don't know, 98 refused]**	

14SAS	**How many of these [*interviewer:* *say number*] children aged 5-12 in this household go to school?**	
	**Number [99 for don't know, 98 refused]:**	

15HOT	**In the last school holiday how many of the [*interviewer:* *say number*] children in your household aged 5-12 (including children not at school) went away and stayed elsewhere for one or more nights?**		
	**Number [99 for don't know, 98 refused]:**		[If zero, skip to 17HGC]

16HOC	**How many of the [*interviewer:* *say number*] children aged 5-12 stayed outside your community?**	
**Number [99 for don't know, 98 refused]:**		

17HGC	**From midnight yesterday to midnight last night, have children aged 5-12 from a different household come and spent time inside your household?**		
	**No**	**Yes**	
	0	1	
	[If no, skip to 19TVO]		

18HGH	**What was the longest time one of these children stayed inside your household?**					
	**less than 5 hrs**	**5-9 hrs**	**10-14 hrs**	**15-19 hrs**	**more than 20 hrs**	**Don't know**
	0	1	2	3	4	99

ACTIVITIES

19TVO	**Do you ever watch TV/video in places other than your household?**					
				**No**	**Yes**	
				0	1	
				[If no, skip to 24MUS]		
20TVF	**In the last week, how many times did you watch TV/video in places other than your household?**					
	**Number (99 for don't know, 98 refused):**				[no skip]	
21TVL	**The last time you did this, how long did you stay there?**					
	**less than 1 hr**	**1-2 hrs**	**3-4 hrs**	**5-6 hrs**	**7-9 hrs**	**More than 10 hrs**
	0	1	2	3	4	5
22TVA	**The last time you did this, how many other adults and youths (those older than 12) were there?**					
**0**	**1-5**	**6-10**	**11-15**	**16-20**	**21-50**	**More than 50**
0	1	2	3	4	5	6

23TVK	**The last time you did this, how many children aged 5-12 were there?**					
**0**	**1-5**	**6-10**	**11-15**	**16-20**	**21-50**	**More than 50**
0	1	2	3	4	5	6

24MUS	**Are you a Muslim?**		
	**No**	**Yes**	
	0	1	

25CHU	**Do you go to church?** [*interviewer: If Muslim, replace ‘church’ with ‘mosque’ for this set of questions*]		
	**No**	**Yes**	
	0	1	
	[If no, skip to 30BAR]		
26CHF	**In the last week, how many times did you go to church** [a mosque]**?**		
	**Number [99 for don’t know, 98 refused]:**		[no skip]

27CHL	**The last time you went to church** [a mosque]**, how long did you stay there?**					
	**less than 1 hr**	**1-2 hrs**	**3-4 hrs**	**5-6 hrs**	**7-9 hrs**	**More than 10 hrs**
	0	1	2	3	4	5
28CHA	**The last time you went to church** [a mosque]**, how many adults and youths (those older than 12) were there?**					
**0**	**1-5**	**6-10**	**11-15**	**16-20**	**21-50**	**More than 50**
0	1	2	3	4	5	6

29CHK	**The last time you went to church** [a mosque]**, how many children aged 5-12 were there?**					
**0**	**1-5**	**6-10**	**11-15**	**16-20**	**21-50**	**More than 50**
0	1	2	3	4	5	6

30BAR	**Do you ever go to bars, discos or shebeens?**		
	**No**	**Yes**	
	0	1	
	[If no, skip to 35HAI]		

31BAF	**In the last week, how many times did you go to a bar, disco or shebeen?**		
	**Number [99 for don’t know, 98 refused]:**		[no skip]

32BAL	**The last time you were in a bar, disco or shebeen, how long did you stay there?**					
	**less than 1 hr**	**1-2 hrs**	**3-4 hrs**	**5-6 hrs**	**7-9 hrs**	**More than 10 hrs**
	0	1	2	3	4	5

33BAA	**The last time you were in a bar, disco or shebeen, how many adults and youths (those older than 12) were there?**					
**0**	**1-5**	**6-10**	**11-15**	**16-20**	**21-50**	**More than 50**
0	1	2	3	4	5	6
34BAK	**The last time you were in a bar, disco or shebeen, how many children aged 5-12 were there?**					
**0**	**1-5**	**6-10**	**11-15**	**16-20**	**21-50**	**More than 50**
0	1	2	3	4	5	6

35HAI	**Do you ever go to a hairdresser or barber?**					
				**No**	**Yes**	
				0	1	
				[If no, skip to 40SCH]		
36HAF	**In the last week, how many times did you go to a hairdresser or barber?**					
	**Number [99 for don't know, refused 98]:**				[no skip]	

37HAL	**The last time you were in a hairdressers' or barber, how long did you stay there?**					
	**less than 1 hr**	**1-2 hrs**	**3-4 hrs**	**5-6 hrs**	**7-9 hrs**	**More than 10 hrs**
	0	1	2	3	4	5
38HAA	**The last time you were in a hairdresser or barber, how many adults and youths (those older than 12) were there?**					
**0**	**1-5**	**6-10**	**11-15**	**16-20**	**21-50**	**More than 50**
0	1	2	3	4	5	6
39HAK	**The last time you were in a hairdresser or barber, how many children aged 5-12 were there?**					
**0**	**1-5**	**6-10**	**11-15**	**16-20**	**21-50**	**More than 50**
0	1	2	3	4	5	6

40SCH	**Do you ever go into school classrooms?**					
			**No**	**Yes**		
			0	1		
			[If no, skip to 45TAX]			
41SCF	**In the last week, how many times did you go into a school classroom?**					
	**Number [99 for don't know, 98 refused]:**			[no skip]		

42SCL	**The last time you were in a school classroom, how long did you stay there?**					
	**less than 1 hr**	**1-2 hrs**	**3-4 hrs**	**5-6 hrs**	**7-9 hrs**	**More than 10 hrs**
	0	1	2	3	4	5
43SCA	**The last time you were in a school classroom, how many adults and youths (those older than 12) were there?**					
**0**	**1-5**	**6-10**	**11-15**	**16-20**	**21-50**	**More than 50**
0	1	2	3	4	5	6
44SCK	**The last time you were in a school classroom, how many children aged 5-12 were there?**					
**0**	**1-5**	**6-10**	**11-15**	**16-20**	**21-50**	**More than 50**
0	1	2	3	4	5	6

TRAVEL

45TAX	**Do you ever use minibuses?**			
		**No**	**Yes**	
		0	1	
		[If no, skip to 52PEO]		

46TXA	**The last time you used a minibus, how many adults and youths (those aged more than 12) were inside the minibus?**			
	**0-5**	**6-10**	**more than 10**	
	0	1	2	

47TXK	**The last time you used a minibus, how many children aged 5-12 were inside the mini-bus?**			
	**0**	**1-5**	**more than 5**	
	0	1	2	

48TXL	**The last time you used a minibus, how long were you inside the minibus?**				
	**less than 10 mins**	**10-29 mins**	**30-59 mins**	**more than 1 hr**	
	0	1	2	3	

49TXF	**During the last week, how many times did you use a minibus?**				
	**Didn’t use**	**1-2**	**3-6**	**more than 6**	
	0	1	2	3	
	[If 0, skip to 52PEO]				

50TXC	**Was anyone coughing on the minibus during the last week?**			
		**No**	**Yes**	
		0	1	
		[If no, skip to 52PEO]		

51TXB	**If so, what things did you and other people on the bus do?**<br>[more than one answer OK]			
	**Sympathise**	0	**Open a window**	3
	**Complain**	1	**Nothing**	4
	**Turn away, or cover nose or mouth**	2		

YESTERDAY'S CONTACTS

52PEO	**I would like to ask you about the people you talked to face-to-face *yesterday*.** We are only interested in the people you had a face-to-face conversation (within arm's reach) with from midnight yesterday to midnight last night. I would like you to include people in your household.<br><br>First, we will try to remember who they were, and I will write down their name (e.g. "Mary") or a description (e.g. "street vendor") that we can use to refer back to them. We will not keep the names – they only need to be detailed enough to help you remember who you meant. Nicknames or initials are fine. If you met the same person on more than one occasion yesterday, I will record them once, and later we will estimate the total time you spent with them.<br>Think about the morning at home: who did you talk to there?<br>Did you go out? Who did you talk to while you were travelling?<br>Did you go out on business? Think about who you talked to there?<br>Did you go shopping? Did you talk to anyone while shopping?<br>Did you go out in the evening? Who did you talk to then?

Person name or description (1)	(This is to aid memory and will not be retained.)										
Number		**01**	02	**03**	04	**05**	06	**07**	08	**09**	10
What was their **age**?	0-4yrs	**0**	0	**0**	0	**0**	0	**0**	0	**0**	0
	5-12 yrs	**1**	1	**1**	1	**1**	1	**1**	1	**1**	1
	13-25 yrs	**2**	2	**2**	2	**2**	2	**2**	2	**2**	2
	26-45 yrs	**3**	3	**3**	3	**3**	3	**3**	3	**3**	3
	Older than 45 yrs	**4**	4	**4**	4	**4**	4	**4**	4	**4**	4
What was their **sex**?	F	**0**	0	**0**	0	**0**	0	**0**	0	**0**	0
	M	**1**	1	**1**	1	**1**	1	**1**	1	**1**	1
Do they **belong** to your:	Household	**0**	0	**0**	0	**0**	0	**0**	0	**0**	0
	Workplace	**1**	1	**1**	1	**1**	1	**1**	1	**1**	1
	Neither	**2**	2	**2**	2	**2**	2	**2**	2	**2**	2
**Where** were you when you were with them most?	Outside	**0**	0	**0**	0	**0**	0	**0**	0	**0**	0
	On transport	**1**	1	**1**	1	**1**	1	**1**	1	**1**	1
	Your home	**2**	2	**2**	2	**2**	2	**2**	2	**2**	2
	Other home	**3**	3	**3**	3	**3**	3	**3**	3	**3**	3
	Shop	**4**	4	**4**	4	**4**	4	**4**	4	**4**	4
	Church	**5**	5	**5**	5	**5**	5	**5**	5	**5**	5
	Bar/disco/shebeen	**6**	6	**6**	6	**6**	6	**6**	6	**6**	6
	School	**7**	7	**7**	7	**7**	7	**7**	7	**7**	7
	Clinic/hospital	**8**	8	**8**	8	**8**	8	**8**	8	**8**	8
	Hairdresser/barber	**9**	9	**9**	9	**9**	9	**9**	9	**9**	9
	Your work building	**10**	10	**10**	10	**10**	10	**10**	10	**10**	10
	Other/ don't know	**11**	11	**11**	11	**11**	11	**11**	11	**11**	11
What was the total **time** you were with them during the day?	Less than 5 mins	**0**	0	**0**	0	**0**	0	**0**	0	**0**	0
	5-14 mins	**1**	1	**1**	1	**1**	1	**1**	1	**1**	1
	15-59 mins	**2**	2	**2**	2	**2**	2	**2**	2	**2**	2
	1-4 hrs	**3**	3	**3**	3	**3**	3	**3**	3	**3**	3
	5-7 hrs	**4**	4	**4**	4	**4**	4	**4**	4	**4**	4
	More than 8 hours	**5**	5	**5**	5	**5**	5	**5**	5	**5**	5
Were you inside or outside the **community** when you were with them most?	Inside	**0**	0	**0**	0	**0**	0	**0**	0	**0**	0
	Outside	**1**	1	**1**	1	**1**	1	**1**	1	**1**	1
	Both	**2**	2	**2**	2	**2**	2	**2**	2	**2**	2
How often do you normally talk to this person?	Never before	**0**	0	**0**	0	**0**	0	**0**	0	**0**	0
	Less than monthly	**1**	1	**1**	1	**1**	1	**1**	1	**1**	1
	1-3 times a month	**2**	2	**2**	2	**2**	2	**2**	2	**2**	2
	1-6 times a week	**3**	3	**3**	3	**3**	3	**3**	3	**3**	3
	Daily	**4**	4	**4**	4	**4**	4	**4**	4	**4**	4

Person name or de-scription (2)	(This is to aid memory and will not be re-tained.)										
Number		**11**	12	**13**	14	**15**	16	**17**	18	**19**	20
What was their **age**?	0-4yrs	**0**	0	**0**	0	**0**	0	**0**	0	**0**	0
	5-12 yrs	**1**	1	**1**	1	**1**	1	**1**	1	**1**	1
	13-25 yrs	**2**	2	**2**	2	**2**	2	**2**	2	**2**	2
	26-45 yrs	**3**	3	**3**	3	**3**	3	**3**	3	**3**	3
	Older than 45 yrs	**4**	4	**4**	4	**4**	4	**4**	4	**4**	4
What was their **sex**?	F	**0**	0	**0**	0	**0**	0	**0**	0	**0**	0
	M	**1**	1	**1**	1	**1**	1	**1**	1	**1**	1
Do they **belong** to your:	Household	**0**	0	**0**	0	**0**	0	**0**	0	**0**	0
	Workplace	**1**	1	**1**	1	**1**	1	**1**	1	**1**	1
	Neither	**2**	2	**2**	2	**2**	2	**2**	2	**2**	2
**Where** were you when you were with them most?	Outside	**0**	0	**0**	0	**0**	0	**0**	0	**0**	0
	On transport	**1**	1	**1**	1	**1**	1	**1**	1	**1**	1
	Your home	**2**	2	**2**	2	**2**	2	**2**	2	**2**	2
	Other home	**3**	3	**3**	3	**3**	3	**3**	3	**3**	3
	Shop	**4**	4	**4**	4	**4**	4	**4**	4	**4**	4
	Church	**5**	5	**5**	5	**5**	5	**5**	5	**5**	5
	Bar/disco/shebeen	**6**	6	**6**	6	**6**	6	**6**	6	**6**	6
	School	**7**	7	**7**	7	**7**	7	**7**	7	**7**	7
	Clinic/hospital	**8**	8	**8**	8	**8**	8	**8**	8	**8**	8
	Hairdresser/barber	**9**	9	**9**	9	**9**	9	**9**	9	**9**	9
	Your work building	**10**	10	**10**	10	**10**	10	**10**	10	**10**	10
	Other/ don’t know	**11**	11	**11**	11	**11**	11	**11**	11	**11**	11
What was the total **time** you were with them during the day?	Less than 5 mins	**0**	0	**0**	0	**0**	0	**0**	0	**0**	0
	5-14 mins	**1**	1	**1**	1	**1**	1	**1**	1	**1**	1
	15-59 mins	**2**	2	**2**	2	**2**	2	**2**	2	**2**	2
	1-4 hrs	**3**	3	**3**	3	**3**	3	**3**	3	**3**	3
	5-7 hrs	**4**	4	**4**	4	**4**	4	**4**	4	**4**	4
	More than 8 hours	**5**	5	**5**	5	**5**	5	**5**	5	**5**	5
Were you inside or outside the **community** when you were with them most?	Inside	**0**	0	**0**	0	**0**	0	**0**	0	**0**	0
	Outside	**1**	1	**1**	1	**1**	1	**1**	1	**1**	1
	Both	**2**	2	**2**	2	**2**	2	**2**	2	**2**	2
How often do you normally talk to this person?	Never before	**0**	0	**0**	0	**0**	0	**0**	0	**0**	0
	Less than monthly	**1**	1	**1**	1	**1**	1	**1**	1	**1**	1
	1-3 times a month	**2**	2	**2**	2	**2**	2	**2**	2	**2**	2
	1-6 times a week	**3**	3	**3**	3	**3**	3	**3**	3	**3**	3
	Daily	**4**	4	**4**	4	**4**	4	**4**	4	**4**	4

53PEP	**How many extra people, beyond those listed above, do you think you talked to yesterday? [**only ask if 20 contacts were listed above, otherwise write 0]		
	**Number [0 if all listed above, 99 for don't know, 98 refused]:**		

54BUH	**Did you leave your home yesterday (i.e. from midnight yesterday to midnight last night)?**			
		**No**	**Yes**	
		0	1	
		[If no, skip to 56JOB]		

55BUI	**I would like to ask you about the buildings you went into *yesterday*.**<br>We are only interested in the buildings that you entered from midnight yesterday to midnight last night.<br>Think about the buildings you entered during the morning (not including your home)?<br>Did you go out on business? What buildings did you go into?<br>Did you go out in the evening? If so, what buildings did you enter?

BUILDING NUMBER		1	2	3	4	5	6	7	8	9	10	11	12	13	14	15	16	17	18	19
What **type** of building did you enter?	Other home	0	0	0	0	0	0	0	0	0	0	0	0	0	0	0	0	0	0	0
	Shop	1	1	1	1	1	1	1	1	1	1	1	1	1	1	1	1	1	1	1
	Church	2	2	2	2	2	2	2	2	2	2	2	2	2	2	2	2	2	2	2
	Bar/disco/shebeen	3	3	3	3	3	3	3	3	3	3	3	3	3	3	3	3	3	3	3
	School	4	4	4	4	4	4	4	4	4	4	4	4	4	4	4	4	4	4	4
	Clinic/hospital	5	5	5	5	5	5	5	5	5	5	5	5	5	5	5	5	5	5	5
	Hairdresser/barber	6	6	6	6	6	6	6	6	6	6	6	6	6	6	6	6	6	6	6
	Your work building	7	7	7	7	7	7	7	7	7	7	7	7	7	7	7	7	7	7	7
	Other	8	8	8	8	8	8	8	8	8	8	8	8	8	8	8	8	8	8	8
How much **time** did you spend in total inside this building?	Less than 5 mins	0	0	0	0	0	0	0	0	0	0	0	0	0	0	0	0	0	0	0
	5-10 mins	1	1	1	1	1	1	1	1	1	1	1	1	1	1	1	1	1	1	1
	11-59 mins	2	2	2	2	2	2	2	2	2	2	2	2	2	2	2	2	2	2	2
	1-4 hrs	3	3	3	3	3	3	3	3	3	3	3	3	3	3	3	3	3	3	3
	5-8 hrs	4	4	4	4	4	4	4	4	4	4	4	4	4	4	4	4	4	4	4
	9-13	5	5	5	5	5	5	5	5	5	5	5	5	5	5	5	5	5	5	5
	More than 14 hrs	6	6	6	6	6	6	6	6	6	6	6	6	6	6	6	6	6	6	6
How many **adults and youths (those older than 12)** were present?	Less than 5	0	0	0	0	0	0	0	0	0	0	0	0	0	0	0	0	0	0	0
	5-9	1	1	1	1	1	1	1	1	1	1	1	1	1	1	1	1	1	1	1
	10-20	2	2	2	2	2	2	2	2	2	2	2	2	2	2	2	2	2	2	2
	Greater than 20	3	3	3	3	3	3	3	3	3	3	3	3	3	3	3	3	3	3	3
How many **children (5-12)** were present?	Less than 5	0	0	0	0	0	0	0	0	0	0	0	0	0	0	0	0	0	0	0
	5-9	1	1	1	1	1	1	1	1	1	1	1	1	1	1	1	1	1	1	1
	10-20	2	2	2	2	2	2	2	2	2	2	2	2	2	2	2	2	2	2	2
	Greater than 20	3	3	3	3	3	3	3	3	3	3	3	3	3	3	3	3	3	3	3
Was this inside your **commu-nity**?	Inside	0	0	0	0	0	0	0	0	0	0	0	0	0	0	0	0	0	0	0
	Outisde	1	1	1	1	1	1	1	1	1	1	1	1	1	1	1	1	1	1	1
Did you visit **once** or more?	Once	0	0	0	0	0	0	0	0	0	0	0	0	0	0	0	0	0	0	0
	More than once	1	1	1	1	1	1	1	1	1	1	1	1	1	1	1	1	1	1	1

WORK

56JOB	**How have you contributed to household living during the past year?** [more than one answer OK]			
	**Working on own land**	0	**Own business**	4
	**Occasional/seasonal employment**	1	**Student**	5
	**Employed**	2	**Housewife/ home-maker**	6
	**No contribution**	3	**Welfare grant**	7

57MIN	**Have you ever worked in a mine?**			
		**No**	**Yes**	
		0	1	
58PRI	**This is a personal question which is important for TB transmission. Would you mind telling us if you have you ever been to prison?** *[interviewer; if necessary reassure them that we are not interested in the reason for their imprisonment]*			
		**No**	**Yes**	**Refused**
		0	1	98

59AWA	**During the last 6 months, how many weeks did you spend away from your household?**	
	**Number [99 if don't know, 98 refused]:**	

ILLNESS/FUNERALS

60FUN	**Have you been to a funeral in the past month?**			
		**No**	**Yes**	
		0	1	
61HOY	**Have you been hospitalized during the last year?**			
		**No**	**Yes**	
		0	1	

62HCS	**Have you attended a healthcare facility in the last 6 months?**			
		**No**	**Yes**	
		0	1	
		[If no, skip to 65HCH]		

63HCC	**In the last 6 months, have you attended a healthcare facility accompanying a child aged 5-12?**			
		**No**	**Yes**	
		0	1	

64HCR	**In the last 6 months, have you attended a healthcare facility to look after a friend or relative?**			
		**No**	**Yes**	
		0	1	

65HCH	**In the last 6 months, have you sent a child aged 5-12 for any reason to a healthcare facility?**			
		**No**	**Yes**	
		0	1	

If you have been ill with a cough for more than 5 days, which of the following would you avoid:		
		**Mark if Avoid**
66CAF	**Seeing friends?**	
67CAT	**Using public transport?**	
68CAW	**Going to work?**	
69CAB	**Visiting bars, discos or shebeens?**	
70CAC	**Going to church?**	
71CAH	**Going to the hairdresser/barber?**	
72CAK	**Contact with children?**	
73CAS	**Going into schools?**	
74CAM	**Going to a clinic?**	
75CAV	**Watching TV/video or playing games somewhere other than your home?**	

In which of the following places do you think children are at risk of catching TB :		
		**Mark for At risk**
76RTC	**TB corners/TB clinics?**	
77RCL	**Clinics (other)?**	
78RSC	**Schools?**	
79RMK	**Markets/shops?**	
80ROH	**Other people's households?**	
81RHH	**Own household?**	
82RCH	**Churches?**	
83RBA	**Bars/discos/shebeens?**	
84RVC	**Games/Video shops, or while watching TV/video somewhere other than at home?**	
85RHB	**Hairdressers/barbers?**	
86RMB	**Minibuses?**	
87ROC	**Outside the community?**	

Thank you very much for your time in helping with this survey. Are there any **questions** that you would like to ask?

88ENT	**End time**				

	Id number (3 digits)	Date [dd/mm/yyyy]	Signature
Research assistant		[RA leave blank]	
Team leader			
Data entry 1			
Data entry 2			

Web Appendix 2

Supporting Methods: Weighting Methodology

The sampling design used was a two-stage, stratified design. The first stage consisted of a sample of standard enumeration areas (SEAs) from within study communities; the second stage consisted of the selection of individuals from within age and gender strata within the SEA. Differences in the SEA size and the age/gender structures meant all eligible participants did not have an equal likelihood of inclusion in the study.

For this reason, a sample weight was calculated for each interviewee. This reflected the inverse probability of that individual being included in the study because of the sampling design, and was used in conjunction with the svyset command in STATA.

The weight was calculated in two stages. A SEA weight was calculated from the probability of an SEA being selected from within a community (i.e. proportional to the number of individuals who consented in the ZAMSTAR final prevalence survey, without replacement). Individual weights were calculated using the number of eligible participants in that interviewee's age and gender stratum within their SEA. The overall weight for each interviewee was a product of both the SEA and individual weights.

Location of contact episodes

The possible locations for close contacts *were 'outside', 'on transport', 'own home', 'other home', 'shop', church', 'bar/disco/shebeen', 'school', 'clinic/hospital', 'hairdresser/barber', 'work building' and 'other'.* The possible locations for casual contacts were *'other home', 'shop', 'church', 'bar/disco/sheeban', 'school', 'clinic/hospital', 'hairdresser/barber', 'work building' and 'other'.*

A community was classed as rural if farming was the livelihood of a substantial proportion of people within the catchment area of its main health facility. For this proportion, population density was also often comparatively low and housing type was often decidedly rural.

Web Table 1

Underlying demography used in Equations 1-3

country	gender	age	population
SA	F	18-25	505
SA	F	26-45	622
SA	F	45+	381
SA	M	18-25	401
SA	M	26-45	522
SA	M	45+	271
ZM	F	18-25	904
ZM	F	26-45	944
ZM	F	45+	538
ZM	M	18-25	1004
ZM	M	26-45	1058
ZM	M	45+	470
SA	F	0-4	280
SA	F	5-12	382
SA	F	13-25	739
SA	F	26-45	622
SA	F	45+	381
SA	M	0-4	258
SA	M	5-12	348
SA	M	13-25	590
SA	M	26-45	522
SA	M	45+	271
ZM	F	0-4	668
ZM	F	5-12	1201
ZM	F	13-25	1752
ZM	F	26-45	944
ZM	F	45+	538
ZM	M	0-4	660
ZM	M	5-12	1216
ZM	M	13-25	1728
ZM	M	26-45	1058
ZM	M	45+	470

Web Appendix 3

Sensitivity to different mixing patterns

As close contact counts will not correlate perfectly with effective contact rates, resulting in different patterns and degrees of age-assortativity, we investigate how different levels of assortativity affect our result that adult infection rates are under-estimated by TB infection rates in children.

We consider a simple model for the proportion of contacts for an individual in group α and group i: $p_{\alpha i}$ (the corresponding data are graphed in the main text, Figure 1).

$$p_{\alpha i} = q.\delta_{\alpha,i+2} + \frac{1}{5}(1-q)$$

That is, a proportion q of an individual's contacts are with a corresponding age group $i+2$ (the advancing peak in main text Figure 1), and a proportion $(1-q)$ of an individual's contacts are at random across the 5 age groups. From this equation, an estimator of q from data on $p_{\alpha i}$ can be computed as: $\hat{q} = 5.(\sum_{\alpha=1}^{3}\sum_{i=3}^{5} p_{\alpha i} - 9)/6$.

We used this contact pattern, together with the demographic and prevalence data for South Africa and Zambia, under the assumption that the overall contact rates for an individual did not depend on the group α, to investigate how the infection rate in the 5-12 year age group underestimated that in adult age groups as the assortativity q changes. For this analysis, we aggregated demography and prevalence across males and females.

Web Figure 1

Proportion (95% confidence interval) of reported (a) close contacts, (b) close contact duration, (c) casual contacts and (d) casual contact duration, by contact location and setting for Zambia and the Western Cape, South Africa, 2011. (Zambia = solid bars; Western Cape, South Africa = dashed bars)

(a)

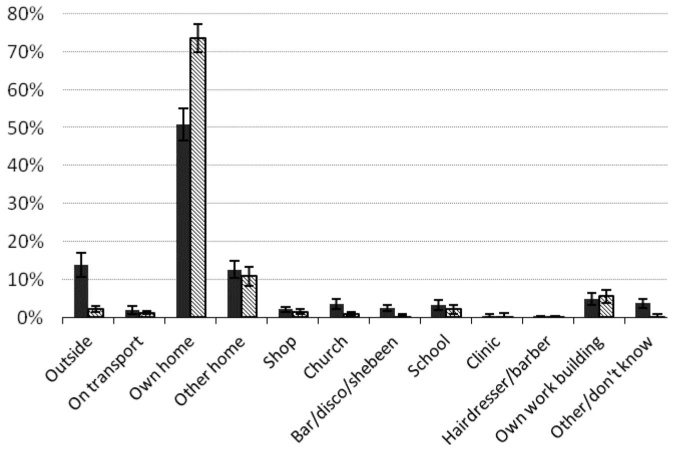

Zambia N=10,380 close contacs, Western Cape, South Africa N=6,637 close contacs. 4,34/17,451 contacts excluded due to missing location data.

(b)

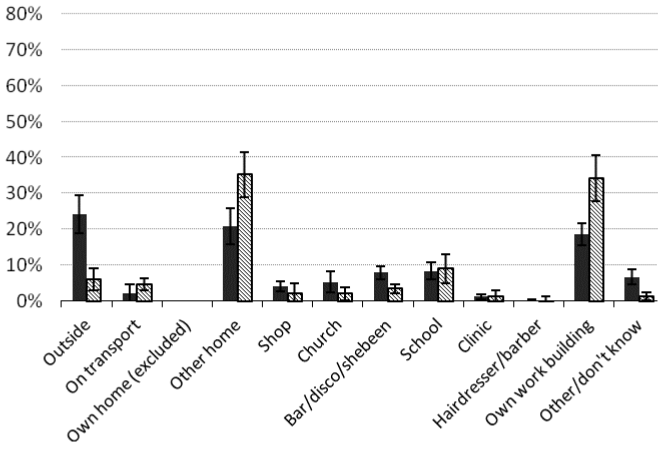

Zambia N = 5,028 close contacs; Western Cape, South Africa N = 1,806 close contacs. 9,986/16,820 'own home' contacts excluded.

(c)

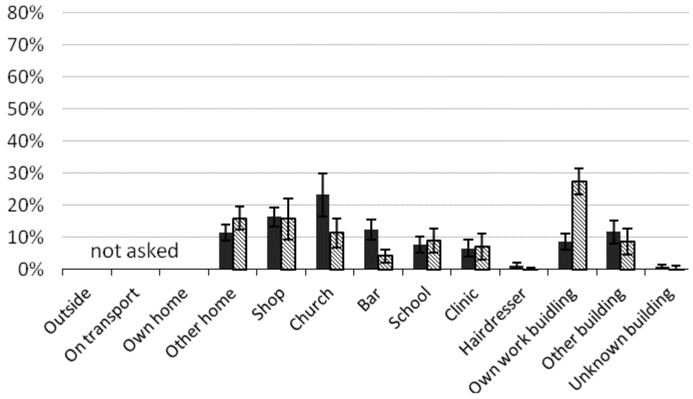

Zambia N=26,331 casual contacts; Western Cape, South Africa N=11,797 casual contacts.

(d)

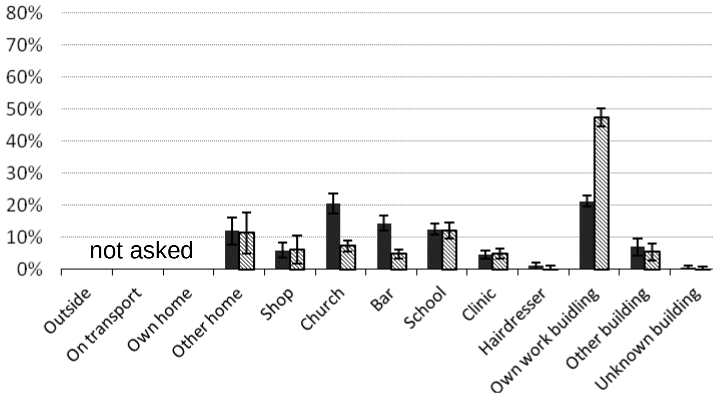

Zambia: 26,182 casual contacts; Western Cape, South Africa: 11,757 casual contacts. 189/38,128 casual contacts excluded due to missing duration data.

Web Table 2

Percentage [95% confidence interval] of close contacts of 18-25 year olds, 26-45 year olds and >45 year olds by gender and setting (rows), with all ages and gender (columns), and their crude total contact rate (final column).

	Gender	Age	With females 0-4	With females 5-12	With females 13-25	With females All ages	With females >45	With males 0-4	With males 5-12	With males 13-25	With males 26-45	With males >45	With males All ages	all ages	Crude total contact rate per day
Western Cape, South Africa	Female	18-25	5.2[4.0;6.5]	7.3[5.9;8.8]	26.9[24.4;29.5]	16.8[14.7;18.9]	8.8[7.2;10.4]	4.9[3.7;6.2]	4.6[3.5;5.9]	9.2[7.6;10.9]	11.0[9.3;12.9]	5.2[4.0;6.6]	35.0[32.4;37.8]	100	5.7[5.3;6.0]
		26-45	5.8[4.7;7.0]	8.3[7.0;9.7]	15.5[13.6;17.3]	23.8[21.7;26.0]	7.3[6.0;8.6]	5.1[4.0;6.2]	5.8[4.7;7.1]	8.9[7.5;10.4]	14.5[12.8;16.3]	4.9[3.9;6.0]	39.3[36.9;41.7]	100	5.4 [5.1;5.6]
		>45	2.9[1.8;4.1]	6.6[4.9;8.4]	16.2[13.6;18.8]	19.4[16.6;22.2]	10.3[8.2;12.5]	4.6[3.1;6.1]	6.2[4.5;7.9]	13.0[10.7;15.5]	11.7[9.4;14.1]	9.2[7.2;11.3]	44.7[41.2;48.2]	100	5.3[4.9;5.7]
	Male	18-25	2.4[1.5;3.4]	3.2[2.2;4.3]	15.5[13.3;17.6]	11.9[10.0;13.9]	8.3[6.6;9.9]	2.2[1.3;3.1]	4.5[3.3;5.8]	30.2[27.5;33.1]	15.0[12.9;17.2]	6.9[5.5;8.5]	58.8[55.7;61.8]	100	5.5[5.2;5.9]
		26-45	2.6[1.8;3.5]	4.2[3.2;5.3]	12.3[10.6;14.1]	16.6[14.6;18.6]	7.0[5.7;8.4]	2.9[2.0;3.8]	4.0[3.0;5.1]	12.4[10.7;14.2]	29.4[27.0;31.8]	8.6[7.1;10.1]	57.3[54.6;59.9]	100	4.7 [4.5;5.0]
		>45	3.7[2.4;5.2]	4.7[3.2;6.3]	11.7[9.5;14.1]	14.4[11.9;17.0]	12.4[10.1;14.9]	3.0[1.9;4.4]	5.8[4.2;7.6]	13.0[10.5;15.5]	18.4[15.6;21.1]	12.9[10.4;15.4]	53.0[49.4;56.6]	100	4.7[4.4;5.1]
Zambia	Female	18-25	2.9[2.1;3.7]	4.9[3.9;5.9]	34.5[32.2;36.7]	17.7[15.9;19.5]	5.2[4.2;6.3]	2.3[1.6;3.0]	4.0[3.1;5.0]	14.3[12.7;16.0]	10.9[9.4;12.4]	3.4[2.5;4.2]	34.9[32.6;37.1]	100	4.4[4.2;4.6]
		26-45	3.4[2.8;4.1]	6.6[5.6;7.5]	16.7[15.3;18.2]	28.9[27.1;30.6]	6.4[5.5;7.3]	2.8[2.2;3.4]	6.4[5.4;7.3]	9.7[8.6;10.9]	14.4[13.1;15.8]	4.6[3.8;5.4]	38.0[36.1;39.8]	100	4.7[4.5;4.9]
		>45	1.3[0.7;2.1]	5.1[3.8;6.5]	17.3[15.1;19.6]	23.5[20.9;25.9]	15.0[12.9;17.1]	2.8[1.8;3.8]	5.3[4.0;6.7]	10.2[8.4;12.0]	8.1[6.5;9.7]	11.4[9.5;13.3]	37.7[34.9;40.7]	100	4.5[4.2;4.7]
	Male	18-25	0.9[0.5;1.3]	2.7[2.0;3.5]	15.8[14.2;17.4]	8.7[7.5;10.0]	3.6[2.8;4.4]	1.1[0.7;1.6]	3.6[2.8;4.4]	44.4[42.2;46.6]	14.6[13.1;16.2]	4.6[3.7;5.5]	68.3[66.2;70.3]	100	5.0[4.8;5.2]
		26-45	2.8[2.1;3.6]	5.0[4.0;6.0]	10.7[9.3;12.2]	14.7[13.1;16.4]	3.1[2.3;3.9]	2.1[1.4;2.7]	4.8[3.9;5.8]	14.6[13.0;16.3]	36.0[33.7;38.2]	6.2[5.1;7.3]	63.7[61.4;65.9]	100	4.9[4.6;5.1]
		>45	1.0[0.5;1.6]	3.6[2.6;4.6]	8.9[7.3;10.5]	11.7[10.0;13.4]	12.0[10.3;13.7]	1.1[0.5;1.6]	4.0[3.0;5.1]	9.5[8.0;11.1]	25.8[23.5;28.2]	22.5[20.2;24.8]	63.0[60.4;65.5]	100	4.2[4.0;4.4]

Web Figure 2

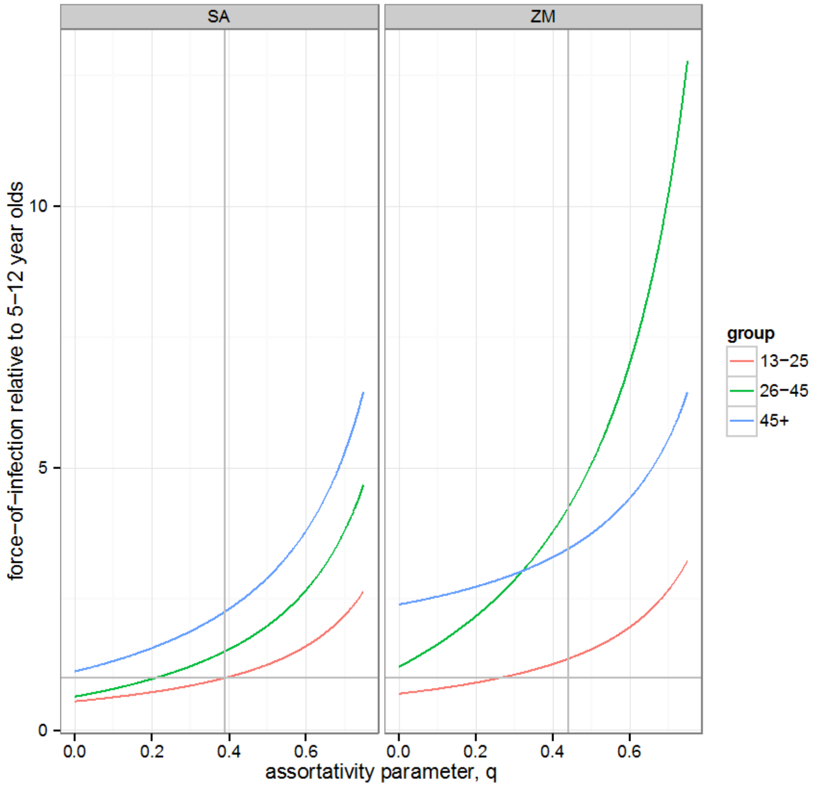

Web Figure 2: The ratio of force of *M.tb* infection in adult age groups to that in the 5-12 year old age group, as a function of age-assortativity in a simple model. The model is defined in the equation above. The vertical grey lines mark the estimate $\hat{q}$ from the South African and Zambian data, and the horizontal grey line is at a ratio of 1.

The assortativity statistics for the South African and Zambian data were 0.39 and 0.44 respectively. For the older age groups, the modelled force-of-infection is substantially higher than that in 5-12 year olds across a wide range of assortativities, particularly in Zambia.

Web Table 3

Model-estimated percentage [95% confidence interval] of total M.tb. infection incidence for each age and gender of susceptible person (columns) due to infectious people (rows) of given age and gender, by setting, for 16 communities in Zambia and 8 communities in Western Cape, South Africa, in 2011.

	Gender	Age	(Susceptible) Female 0-4	5-12	13-25	26-45	>45	(Susceptible) Male 0-4	5-12	13-25	26-45	>45	M+F All ages
Western Cape, South Africa	(Infectious) Female	18-25	18.1[13.9;22.8]	17.2[13.7;20.9]	22.3[19.9;24.8]	12.6[10.9;14.5]	12.7[10.3;15.3]	17.4[13.2;21.9]	11.8[8.8;15.2]	8.5[6.9;10.1]	7.5[6.3;8.9]	8.1[6.1;10.3]	
		26-45	23.9[19.1;28.9]	22.8[19.1;26.9]	15.0[13.0;17.0]	20.9[18.7;23.3]	12.2[10.0;14.6]	21.0[16.6;25.7]	17.3[13.8;21.1]	9.5[8.0;11.2]	11.6[10.1;13.3]	8.9[7.0;10.9]	
		>45	9.6[6.0;13.6]	14.6[10.9;18.6]	12.8[10.6;15.1]	13.9[11.8;16.1]	14.1[11.1;17.3]	15.5[10.8;20.5]	14.9[11.1;19.2]	11.4[9.3;13.7]	7.6[6.1;9.3]	13.5[10.6;16.6]	
	(Infectious) Male	18-25	8.6[5.5;12.0]	7.5[5.1;10.1]	12.9[10.9;15.0]	9.0[7.4;10.7]	12.0[9.5;14.6]	7.7[4.8;11.0]	11.4[8.4;14.8]	27.8[24.7;31.2]	10.3[8.6;12.0]	10.7[8.3;13.3]	
		26-45	20.3[14.5;26.3]	21.5[16.8;26.4]	22.5[19.4;25.6]	27.4[24.3;30.6]	22.1[18.3;26.4]	22.3[16.3;28.3]	22.5[17.4;27.8]	25.0[21.6;28.5]	44.2[40.7;47.7]	29.1[24.7;33.8]	
		>45	19.5[13.2;26.0]	16.4[11.5;21.6]	14.6[11.7;17.7]	16.2[13.2;19.3]	26.9[22.2;31.7]	16.2[10.2;22.6]	22.0[16.4;28.0]	17.8[14.5;21.5]	18.8[15.7;22.0]	29.6[24.5;34.8]	
		All M	48.4[41.6;54.8]	45.4[40.1;50.6]	50.0[47.0;52.8]	52.6[49.7;55.3]	60.9[56.8;64.9]	46.0[39.2;52.4]	56.0[50.4;61.3]	70.6[67.9;73.3]	73.2[71.0;75.4]	69.5[65.5;73.3]	57.3[56.3;58.2]
	M+	All ages	100.0	100.0	100.0	100.0	100.0	100.0	100.0	100.0	100.0	100.0	
Zambia	(Infectious) Female	18-25	14.0[10.2;18.2]	12.0[9.5;14.8]	26.9[24.8;29.2]	12.7[11.2;14.2]	12.4[9.9;15.1]	13.3[9.4;17.9]	10.1[7.7;12.7]	10.0[8.8;11.3]	5.1[4.4;5.9]	5.9[4.4;7.5]	
		26-45	21.6[16.7;26.8]	21.1[17.7;24.6]	17.1[15.4;18.9]	27.0[24.9;29.3]	20.0[17.0;23.2]	21.4[16.3;27.0]	20.9[17.5;24.5]	8.9[7.8;10.1]	8.9[7.9;9.9]	10.6[8.7;12.7]	
		>45	1.9[1.0;3.0]	3.8[2.7;4.9]	4.0[3.4;4.7]	5.0[4.3;5.7]	10.7[8.9;12.6]	4.9[3.1;6.9]	4.0[2.9;5.1]	2.1[1.7;2.5]	1.1[0.9;1.4]	6.0[4.9;7.2]	
	(Infectious) Male	18-25	4.5[2.5;6.9]	7.0[5.2;9.0]	12.9[11.5;14.5]	6.5[5.6;7.6]	9.1[7.0;11.2]	6.8[4.0;9.9]	9.3[7.2;11.7]	32.5[30.0;35.1]	7.2[6.3;8.2]	8.4[6.7;10.2]	
		26-45	55.1[46.9;62.8]	50.8[44.9;56.3]	34.7[31.3;38.1]	43.6[40.3;46.8]	30.3[24.3;36.3]	49.8[40.4;58.3]	49.6[43.6;55.3]	42.3[39.0;45.7]	70.2[67.9;72.3]	44.8[39.6;49.7]	
		>45	2.9[1.4;4.7]	5.4[3.9;7.1]	4.2[3.4;5.1]	5.1[4.3;6.1]	17.5[14.6;20.6]	3.8[1.9;6.0]	6.2[4.5;8.1]	4.1[3.3;4.9]	7.5[6.5;8.5]	24.3[21.2;27.7]	
		All M	62.5[55.3;68.9]	63.2[58.4;67.8]	51.9[49.1;54.6]	55.2[52.5;57.8]	56.9[52.3;61.2]	60.4[52.5;67.6]	65.1[60.4;69.6]	79.0[77.3;80.6]	84.8[83.6;86.1]	77.4[74.6;80.1]	65.7[64.4;66.8]
	M+F	All ages	100.0	100.0	100.0	100.0	100.0	100.0	100.0	100.0	100.0	100.0	
